# A Rare Presentation of Cerebrovascular Accident in a COVID-19 Patient: A Case Report

**DOI:** 10.7759/cureus.12287

**Published:** 2020-12-25

**Authors:** Ross Sattler, Lakmal S Ekanayake, Amber Richardson

**Affiliations:** 1 Medicine, Ohio University Heritage College of Osteopathic Medicine, Athens, USA; 2 Internal Medicine, Springfield Regional Medical Center, Springfield, USA

**Keywords:** sars-cov-2 (severe acute respiratory syndrome coronavirus -2)

## Abstract

Severe acute respiratory syndrome coronavirus 2 (SARS-CoV-2) is a single-stranded, positive-sense, enveloped ribonucleic acid (RNA) virus. SARS-CoV-2 and its associated disease coronavirus disease 2019 (COVID-19) has caused a global pandemic in the year 2019-2020. COVID-19 has caused widespread death, economic burden, and overcrowding of hospitals. As of September 2020, there is no reliable pharmacological treatment for patients affected by COVID-19. Herein we present a case of a 41-year-old Caucasian female who presented to the emergency department with flu-like symptoms for the past five days. The patient was admitted for COVID-19 symptoms and subsequently developed COVID-19 associated thrombotic syndrome and ischemic stroke. Below, we discuss risk factors, pathology, and rare manifestations resulting from COVID-19 infection. COVID-19 primarily affects the lungs, but a review of the current literature shows limited cases of ischemic stroke and diffuse thrombosis induced by infection of the novel COVID-19 in relatively healthy individuals with minimal risk factors.

## Introduction

Severe acute respiratory syndrome coronavirus (SARS-CoV-2) and its disease, coronavirus disease 2019 (COVID-19), is a respiratory infection with potential multisystem involvement that has caused a global pandemic. A unique aspect of this virus is the ability to alter the metabolism of clotting factors, resulting in coagulopathy. The lack of knowledge and novelty of COVID-19 has provided a difficult task for physicians to manage. 

The etiology of COVID-19 is unknown and has caused a significant number of cases worldwide. Mortality rates as high as 2-4% have been reported. SARS-CoV-2 belongs to the family of Coronaviridae and is a positive-sense, enveloped single-stranded, ribonucleic acid (RNA) virus. The etiology of the virus appears to be zoonotic and its origin is uncertain. In humans, SARS-CoV-2 is primarily transmitted via the spread of respiratory droplets due to coughing, sneezing, or talking [1].

The most common symptomology of COVID-19 consists of fever, cough, shortness of breath, myalgias, and loss of taste or smell. Some of the less common presenting signs include pharyngitis, headache, and GI symptoms such as diarrhea [1]. Certain risk factors that increase the susceptibility of the COVID-19 virus include individuals with advanced age and comorbid conditions [2,3]. These include diabetes mellitus, chronic lung disease, cardiovascular disease, obesity, and immunocompromised status [2,3]. These comorbid conditions have been associated with increased hospitalization and intensive care unit admission when compared to non-hospitalized patients [2,3]. 

Current diagnostics and screening protocols remain largely focused on individuals exhibiting symptoms such as fever, cough, and shortness of breath along with asymptomatic individuals with known or suspected exposure [1]. The main two laboratory tests which can identify SARS-CoV-2, are centered around the multiplex assay and the nCOV reverse transcription-polymerase chain reaction diagnostic panel [1].

Current complications from COVID-19 are vast and diverse. Some of these include serious neurological complications, acute respiratory distress syndrome, hematological abnormalities, and altered cranial nerve functions such as anosmia [1,4]. Significant multi-system complications include a mix of coagulopathy with a pro-thrombotic state and neurological complications [5]. Clinical presentation manifests with a significantly increased D-dimer, fibrin/fibrinogen degradation products, and mild thrombocytopenia [5,6]. Notable complications include stroke and cerebrovascular accidents. Importantly, some patients who experienced severe thrombotic events are younger individuals with no known risk factors [4].

The extensive clinical manifestations and their unpredictable outcomes are the most salient concern for COVID-19. By investigating risk factors, commonalities in clinical presentation, and predictors of prognosis, physicians will be able to improve patient management through the development of standard protocols and guidelines for patients who are infected by COVID-19.

## Case presentation

**Figure 1:**
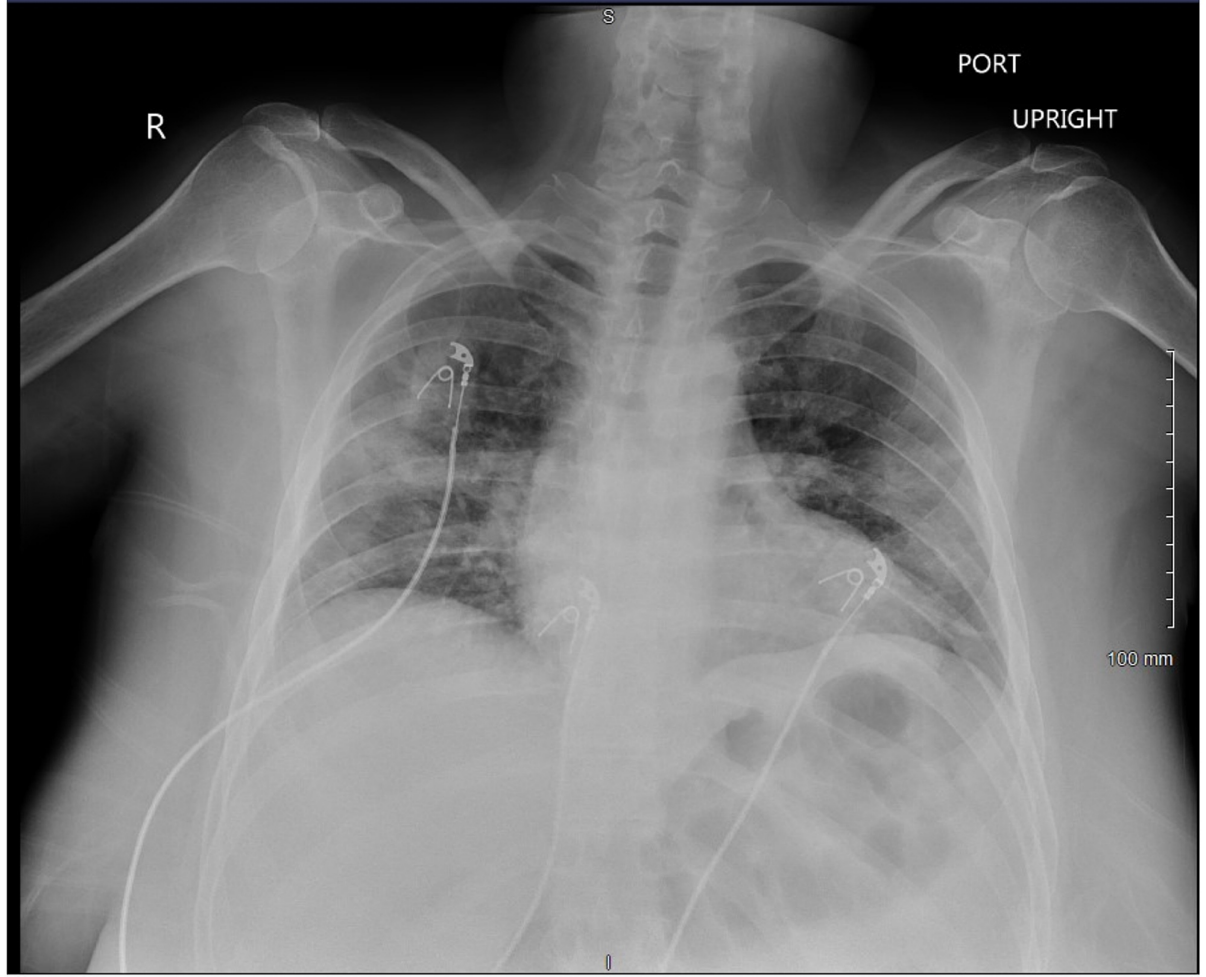
Initial chest x-ray on presentation to the emergency department Chest x-ray showcasing patchy ground-glass opacifications

A 41-year-old Caucasian female presented to the emergency department with a chief complaint of worsening shortness of breath. She tested positive for COVID-19 nine days prior to admission after developing headaches and rhinorrhea. Her symptoms worsened and she was evaluated in the emergency department five days prior to admission with complaints of shortness of breath, cough, fever, nausea, and vomiting. A chest radiograph was taken at this time (Figure 1).

**Figure 2:**
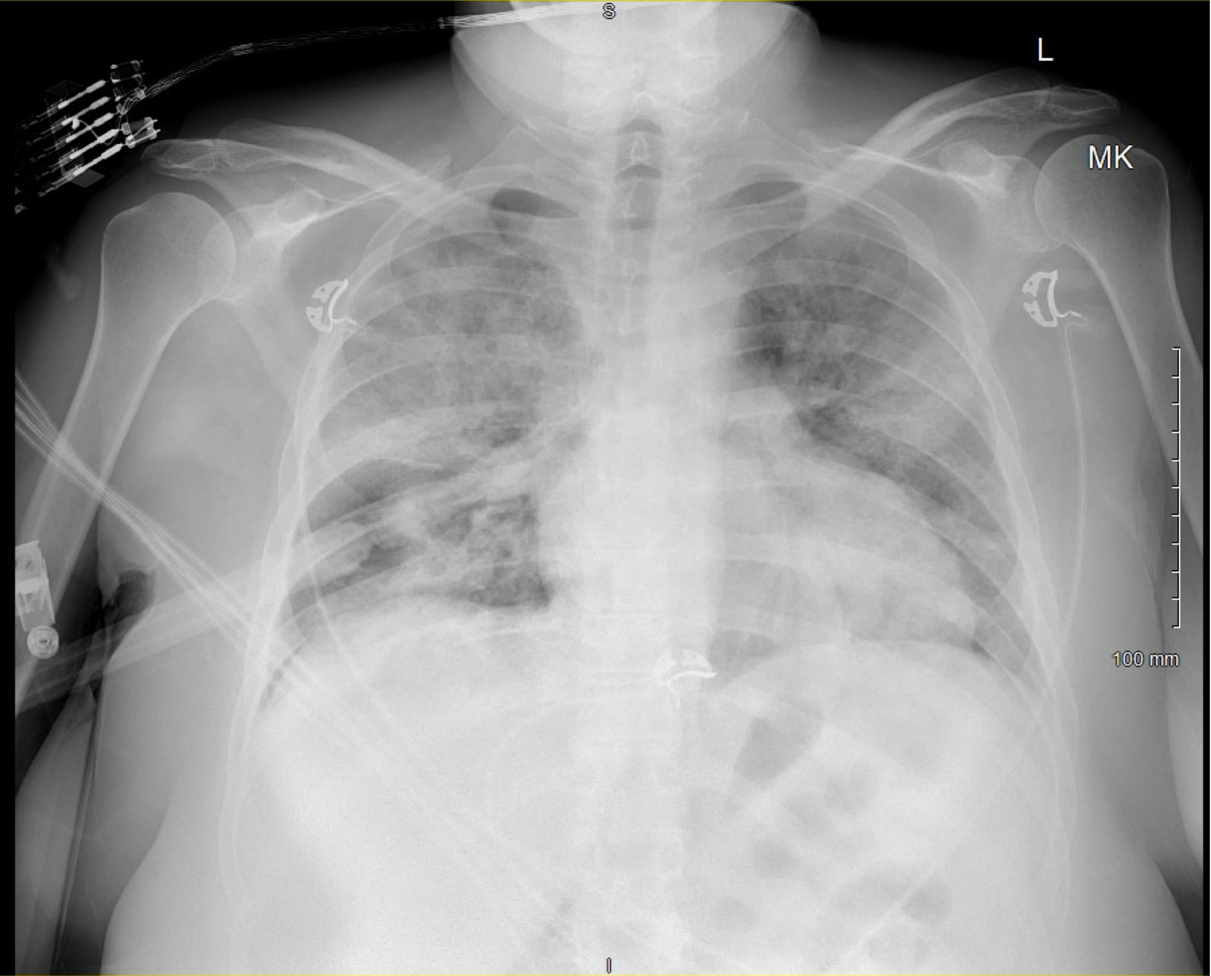
Repeat chest x-ray on the second presentation to the emergency department Repeat chest x-ray showcasing patchy airspace opacities diffusely to both lungs

At this time, she was discharged home with doxycycline and advised to continue to self-isolate. She continued to decompensate at home. On the day of admission, she presented to the emergency department in significant respiratory distress. A repeat chest radiograph was taken at this time (Figure 2). 

Significant past medical history includes rheumatoid arthritis and obesity with a BMI of 31. Current medications include 100mg hydroxychloroquine and 7.5mg prednisone for the management of rheumatoid arthritis. 

In the emergency department, the patient was hypoxemic with an oxygen saturation of 78% and was started on bilevel positive airway pressure (BiPAP). A physical exam revealed diffuse rhonchi in the bilateral lungs. The patient was tachycardic and febrile meeting sepsis criteria and was started on intravenous fluids, ceftriaxone, and azithromycin. Neurological examination revealed no focal deficits. She was admitted for COVID-19-positive acute respiratory failure with hypoxemia and sepsis treatment.

Upon admission, she received 100mg remdesivir. Due to worsening respiratory status and progressive respiratory failure, the patient was intubated and placed in a RotoProne bed on day 2 of hospitalization. Examination of the patient on hospital day 4 revealed new-onset petechiae covering parts of her upper back and bilateral upper arms with noticeable patchy bruising. A complete blood count (CBC) revealed her platelets dropped to 60 and hemoglobin dropped to 10. Coagulation labs showed D-dimer >5250, fibrinogen 161, activated partial thromboplastin time (aPTT) 41.6, and prothrombin time (PT) 14.6. Peripheral blood smear showed no schistocytes. 

To manage thromboembolic complications associated with COVID-19, 300mg aspirin suppository was administered starting hospital day 4. Enoxaparin 40mg was later added to this regimen beginning hospital day 7 in addition to the discontinuation of remdesivir due to the completion of therapy.

**Figure 3:**
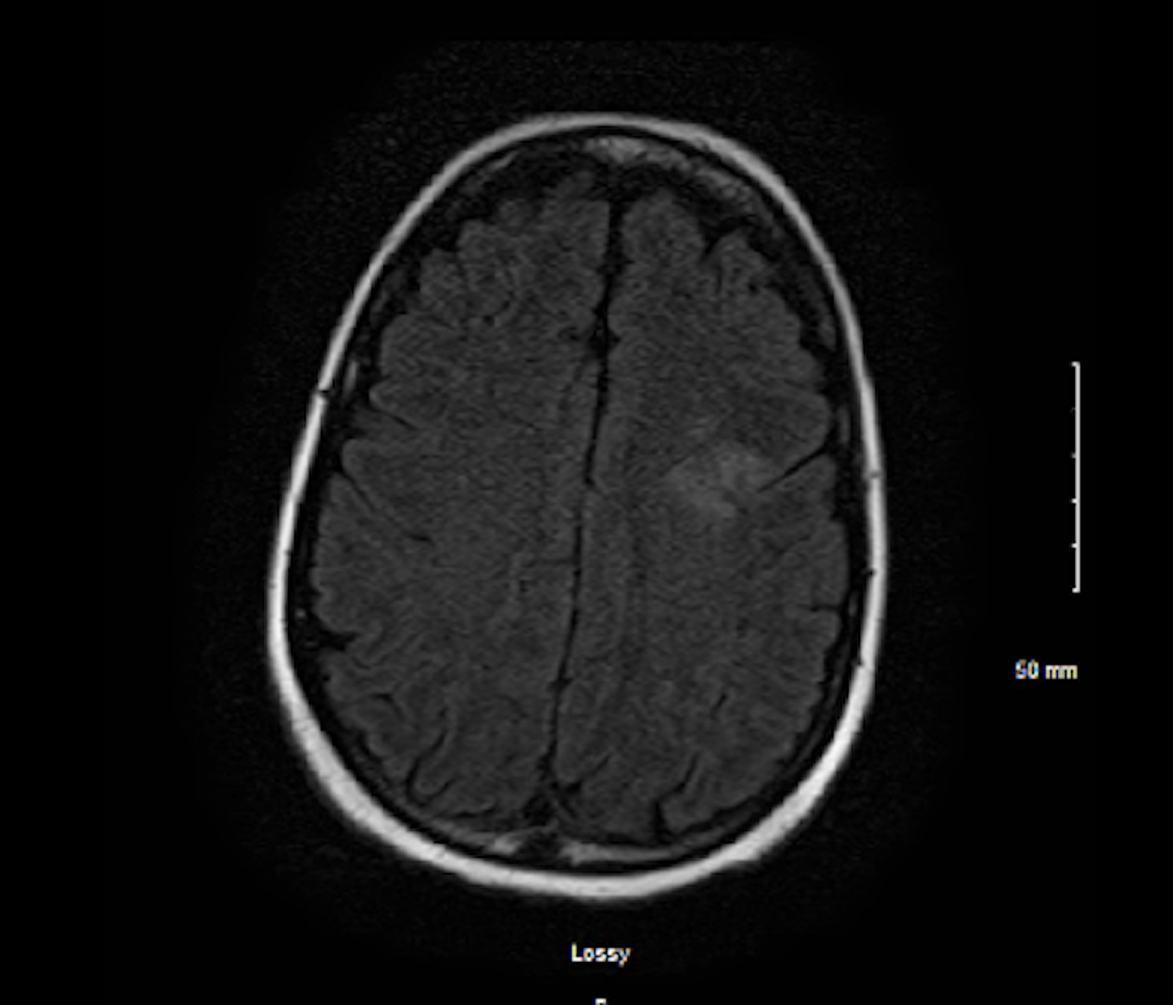
MRI of brain without contrast Magnetic Resonance Imaging revealing an early acute ischemic infarct in the left frontal lobe.

On hospital day 9, due to erratic changes in blood pressure, there was a concern for intracranial pathology. Magnetic resonance imaging (MRI) of the head was performed (Figure 3).

Enoxaparin dose was increased to 80mg. Extubation occurred on day 12 of hospitalization. Subsequent neurological examination revealed spontaneous eye-opening, however, she did not follow commands but did withdraw to pain. She had considerable recovery of neurological function in the following days but significant right hemiparesis remained. She was discharged to an acute rehab facility. 

## Discussion

While current evidence surrounding COVID-19 prognosis is limited, preliminary data have suggested certain clinical biomarkers have been associated with a critical illness. C reactive protein >200, oxygen saturation <88% at admission, and D-dimer level >2,500 were found to have a stronger association with severe illness compared to age or presence of comorbidities [4]. Recent evidence has showcased the significant role of hyper-inflammation, which has been positively correlated with illness severity in patients with COVID-19 infection [7].

One of the theories that best represents the development of thrombosis can be explained using Virchow's triad which describes three major contributors to clot formation. These include endothelial injury, stasis of blood flow, and alterations in factors involved in the coagulation cascade leading to a hypercoagulable state [8]. Currently, the evidence surrounding COVID-19 pathogenesis involving thrombosis is limited. Tissue specimens from critically ill COVID-19 patients have found elevated complement deposits and leukocytes in areas of microvascular injury suggesting inflammatory mediated endothelial injury [9]. Hospitalized patients, especially those who are intubated, are known to be at risk for blood flow stasis due to long periods of immobility. Furthermore, COVID-19 has been shown to have multiple effects on the coagulation cascade which are primarily reflected in abnormal laboratory values such as elevated factor VIII, fibrinogen, D-dimer, von Willebrand factor antigen, and antiphospholipid antibodies; all of which potentiate a prothrombotic state [10,11]. Prolonged coagulation time is not ubiquitously present in patients with COVID-19 and reports of coagulation time have varied [5].

There have been reports of ischemic stroke occurring in patients with COVID-19. Significantly, some cases have been in adults younger than 50 years old with no associated cardiovascular risk factors [4]. Despite reports of ischemic stroke in relatively young and healthy infected patients, the mean age for ischemic stroke was found to be 63.4 years old and associated with significant cardiovascular risk factors [12]. One meta-analysis study estimated the incidence of acute ischemic stroke in COVID-19 patients to be between 0.9-2.7% with a mortality rate of 38%. There was a higher incidence of ischemic stroke in patients with severe COVID-19 illness with a mean presentation of stroke roughly 10 days after the onset of COVID-19 symptoms. This supports the hypothesis that cytokine storm induced by COVID-19 potentially mediates the prothrombotic state. Elevated D-dimer, fibrinogen, and the presence of antiphospholipid antibodies were found in COVID-19 patients with acute ischemic stroke [12]. Further study and data collection are necessary to draw conclusions on the stroke mechanism induced by COVID-19 especially as cases are being reported in younger adults without traditional risk factors for ischemic stroke.

Hypercoagulability is a severe complication that occurs in patients with COVID-19. There is an increasing number of cases reporting a novel thrombotic syndrome associated with COVID-19. This syndrome appears to emulate disseminated intravascular coagulation (DIC)-like clinical picture with mild thrombocytopenia and elevated fibrinogen levels [5]. However, it has been noted this COVID-19 associated thrombotic syndrome carries a heavy predisposition towards thrombosis and does not present as a typical DIC. Notable differences include both bleeding and thrombosis with a low fibrinogen level in DIC [5,13]. Importantly, our patient had two documented fibrinogen levels throughout her hospitalization, both of which were low. In fact, emerging evidence has suggested the thrombotic syndrome associated with COVID-19 may be similar to complement-mediated thrombotic microangiopathy (TMA) syndromes and in particular, antiphospholipid syndrome (APS) or atypical hemolytic uremic syndrome (aHUS) [5].

The primary pathogenesis of TMAs is driven by profound complement activation resulting in thrombotic events with very rare associated bleeding episodes [5]. Preliminary studies have found antiphospholipid antibodies (aPL) in severely ill COVID-19 patients with strong associations with elevated D-dimer and thrombosis [14]. However, this is not uncommon as aPL has known to be associated with viral sepsis [5]. aPL is a pathogenic autoantibody found in APS which causes marked thrombosis [5]. Our patient was not tested for aPL during her hospital course nor could we find medical records indicating she had been tested in the past. Further data is required to draw conclusions on the role of aPL and COVID-19 thrombotic pathogenesis. Based on preliminary data, additional coagulation findings in COVID-19 associated thrombotic syndrome have reported the absence of schistocytes on peripheral blood smear [5]. Notably, our patient did not have any schistocytes present on a blood smear. COVID-19 associated thrombotic syndrome exhibits stark commonalities to APS or aHUS with significant complement manifestation, lack of hemolytic anemia, and both small and large vessel thrombotic involvement [5]. However, lack of data due to the novelty of COVID-19 limits our conclusive scope, and additional studies are required to further this investigation. 

Owing to the novelty of the prothrombotic state induced by COVID-19, management can be challenging for clinicians. Anticoagulation recommendations for both prophylaxis and therapy are currently being developed, however, due to limited data, there are no universal guidelines. Thromboprophylaxis with low molecular weight heparin (LMWH) is recommended for intubated COVID-19 patients in the intensive care unit as this has shown evidence of improved survival [15]. Furthermore, there is some evidence suggesting Enoxaparin improves prognosis especially in COVID-19 patients with elevated D-dimer [16]. In addition to anticoagulation, LMWH also exhibits anti-inflammatory properties and may have added benefit in the hyper-inflammatory state observed in COVID-19 patients [17]. Many providers are using thrombolytics such as tPA per usual indications in ischemic stroke and preliminary evidence has suggested it is both efficacious and safe to use [18].

Outcomes and prognosis for COVID-19 infections in rheumatic patients are currently being investigated and are poorly understood. There is a concern for this population of patients due to their immunocompromised status. The American College of Rheumatology has created a task force to follow the rapidly evolving data and will continue to update its guidelines for the management of rheumatic patients as new evidence emerges [19].

Our patient was on chronic hydroxychloroquine therapy for the treatment of rheumatoid arthritis. Notably, hydroxychloroquine therapy was held upon initiation of remdesivir therapy. There has been debate on the use of hydroxychloroquine and its outcomes in COVID-19 infection. In a review of the current literature, there is no conclusive pharmacologic treatment regimen that improves mortality in patients with COVID-19. Clinical trials with hydroxychloroquine have not provided evidence to support its efficacy in prophylaxis in patients with COVID-19 [20].

## Conclusions

COVID-19 infection has induced a worldwide pandemic that has affected thousands of individuals of numerous age groups, risk factors, and medical histories. Infection of COVID-19 in young, relativity healthy individuals manifests as a mild and transient respiratory illness. An associated systemic thrombotic syndrome leading to a cerebrovascular accident is a rare and unique COVID-19 manifestation that can potentially lead to severe disability and death. 
